# Supplementary material for: Impairment-based assessments for patients with lateral ankle sprain: A systematic review of measurement properties
Source: PLoS One. 2023 Feb 22;18(2):e0280388. doi: 10.1371/journal.pone.0280388 (PMC9946235; doi:10.1371/journal.pone.0280388)
Supplement: S1 Table — Search terms for all six databases. (DOCX) [file pone.0280388.s002.docx]

## S1 Appendix. Search strategies

**PubMed (Search date 01/07/2022)**

("ankle"[MeSH Terms] OR "ankle joint"[MeSH Terms] OR "lateral ankle sprain"[All Fields] OR "Ankle Injuries"[MeSH Terms] OR "ankle sprain"[All Fields] OR ("ankle"[All Fields] AND "sprain"[All Fields]) OR "lateral ligament, ankle"[MeSH Terms] OR "chronic ankle instability"[All Fields]) AND ("diagnosis"[MeSH Terms] OR "assessment"[All Fields] OR "test"[All Fields] OR "patient reported outcome measurements"[All Fields] OR "PROMs"[All Fields] OR "questionnaire"[All Fields]) AND ("clinimetric propert*"[All Fields] OR "clinimetric"[All Fields] OR "validity"[All Fields] OR "criterion validity"[All Fields] OR "construct validity"[All Fields] OR "validation studies as topic"[MeSH Terms] OR "reliability"[All Fields] OR "sensitivity and specificity"[MeSH Terms] OR "responsiveness"[All Fields] OR "minimal important change"[All Fields] OR "minimal clinical difference"[All Fields] OR "Reproducibility of Results"[MeSH Terms] OR "minimal clinically important difference"[MeSH Terms] OR "psychometrics"[MeSH Terms] OR "likelihood ratio"[All Fields])

**2138 results**

**CINAHL (Search date 01/07/2022)**

(MH "ankle" OR MH "ankle joint" OR MH "Ankle Injuries” OR "ankle sprain" OR ("ankle" AND "sprain") OR MH "lateral ligament, ankle" OR "chronic ankle instability") AND

(MH "diagnosis" OR "assessment" OR "test" OR "patient reported outcome measurements" OR "PROMs" OR "questionnaire") AND ("clinimetric” OR "validity” OR "validation studies as topic” OR “reliability” OR MH “sensitivity and specificity” OR “responsiveness” OR MH “reproducibility of results” OR MH “psychometrics” OR “likelihood ratio)

**869 results**

**Ovid Embase (Search date 01/07/2022)**

("ankle".sh. OR "ankle joint".sh. OR "lateral ankle sprain".af. OR "Ankle Injuries".sh. OR “ankle sprain".af. OR "lateral ligament, ankle”.af. OR ("ankle" AND "sprain").af. OR "chronic ankle instability".af.) AND ("diagnosis".sh. OR "assessment".af. OR "test”.af. OR "patient reported outcome measurements”.af. OR "PROMs".af. OR "questionnaire".af.) AND ("clinimetric".af. OR "validity".af. OR "construct validity".af. OR "reliability".af. OR "sensitivity and specificity".sh. OR "responsiveness".af. OR "minimal important change".af. OR "minimal clinical difference".af. OR "Reproducibility of Results".sh. OR "minimal clinically important difference".sh. OR "psychometrics".sh. OR "likelihood ratio".af.)

**993 results**

**Web of Science (Search date 01/07/2022)**

“Ankle” OR “ankle joint” OR “ankle injuries” OR “ankle sprain” OR “lateral ankle sprain” OR (ankle AND sprain) OR “lateral ankle ligament” OR “chronic ankle instability” AND

"diagnosis" OR "assessment" OR "test" OR "patient reported outcome measurements" OR "PROMs" OR "questionnaire" AND “clinimetric” OR "Validity" OR "criterion validity" OR "construct validity" OR "validation studies as topic" OR "reliability" OR "sensitivity and specificity” OR "responsiveness" OR "minimal important change" OR "Reproducibility of Results" OR "minimal clinically important difference" OR "psychometrics" OR "likelihood ratio"

**517 results**

**Cochrane Library (Search date 01/07/2022)**

("ankle joint” OR "lateral ankle sprain" OR "Ankle Injuries” OR "ankle sprain" OR ("ankle" AND "sprain") OR "lateral ligament, ankle" OR "chronic ankle instability") AND ("diagnosis" OR "assessment” OR "test" OR "patient reported outcome measurements” OR "PROMs" OR "questionnaire") AND ("clinimetric” OR "validity" OR "criterion validity” OR "construct validity” OR "validation studies as topic” OR "reliability” OR "sensitivity and specificity” OR "responsiveness” OR "minimal important change” OR "minimal clinical difference” OR "minimal clinically important difference” OR "psychometrics” OR "likelihood ratio”)

**8 Cochrane reviews**

**280 trials**

**SPORTDiscus (Search date 01/07/2022)**

("ankle joint" OR "lateral ankle sprain" OR "Ankle Injuries" OR "ankle sprain" OR ("ankle" AND "sprain") OR "lateral ligament, ankle" OR "chronic ankle instability") AND

("diagnosis" OR "assessment" OR "test" OR "patient reported outcome measurement" OR “patient reported outcome measurements” OR "PROMs" OR "questionnaire")

AND ("clinimetric propert*" OR "clinimetric" OR "validity" OR "validation studies as topic" OR "reliability" OR "sensitivity and specificity" OR "responsiveness" OR "minimal important change" OR "Reproducibility of Results" OR "minimal clinically important difference" OR "psychometrics" OR "likelihood ratio")

**154 results**
