# Supplementary material for: Impairment-based assessments for patients with lateral ankle sprain: A systematic review of measurement properties
Source: PLoS One. 2023 Feb 22;18(2):e0280388. doi: 10.1371/journal.pone.0280388 (PMC9946235; doi:10.1371/journal.pone.0280388)
Supplement: S2 Table — (DOCX) [file pone.0280388.s003.docx]

## S2 Appendix. List of excluded studies during full-text screening

| **Reasons for exclusion** | **Amount** | **Study** |
| --- | --- | --- |
| **Wrong population** | 48 | [100-147] |
| **Cross-cultural adaption studies** | 21 | [148-168] |
| **Wrong outcome** | 13 | [169-181] |
| **Wrong publication type** | 2 | [182, 183] |
| **Wrong study design** | 2 | [184, 185] |
| **No full-text accessible** | 2 | [186, 187] |
| **No full-text published** | 2 | [188, 189] |

100. Aitkenhead I. Ankle joint dorsiflexion assessment: the development of a new weight-bearing method. BR J PODIATRY. 2002;5(2):32-5. PubMed PMID: rayyan-152988646.

101. Amacker A, Meng J, Jordan K, Mathieu N, Sattelmayer M, Spring H, et al. Responsiveness of the Star Excursion Balance Test on Firm and Unstable Underground. Schweizerische Zeitschrift für Sportmedizin & Sporttraumatologie. 2015;63(2):24-8. PubMed PMID: rayyan-666947593.

102. Anaforoglu Kulunkoglu B, Celik D. Reliability and Validity of the Turkish Version of Foot and Ankle Ability Measure for Patients With Chronic Ankle Disability. J Foot Ankle Surg. 2019;58(1):38-41. PubMed PMID: rayyan-152988596.

103. Calvo-Guisado MJ, Díaz-Borrego P, Garcia de Velasco JG, Fernández-Torrico JM, Conejero-Casares JA. Three techniques for measuring dorsal flexion of the ankle: intra- and inter-observer reliability. REHABILITACION. 2007;41(5):200-6. PubMed PMID: rayyan-152988647.

104. Chisholm MD, Birmingham TB, Brown J, MacDermid J, Chesworth BM. Reliability and Validity of a Weight-Bearing Measure of Ankle Dorsiflexion Range of Motion. PHYSIOTHER CAN. 2012;64(4):347-55. PubMed PMID: rayyan-152988573.

105. Cöster MC, Bremander A, Rosengren BE, Magnusson H, Carlsson A, Karlsson MK. Validity, reliability, and responsiveness of the Self-reported Foot and Ankle Score (SEFAS) in forefoot, hindfoot, and ankle disorders. Acta Orthop. 2014;85(2):187-94. PubMed PMID: rayyan-152988548.

106. Cöster MC, Rosengren BE, Bremander A, Brudin L, Karlsson MK. Comparison of the Self-Reported Foot and Ankle Score (SEFAS) and the American Orthopedic Foot and Ankle Society Score (AOFAS). Foot Ankle Int. 2014;35(10):1031-6. PubMed PMID: rayyan-152988642.

107. Cox RW, Martinez RE, Baker RT, Warren L. Validity of a Smartphone Application for Measuring Ankle Plantar Flexion. J Sport Rehabil. 2018;27(3). PubMed PMID: rayyan-152988632.

108. Croy T, Koppenhaver S, Saliba S, Hertel J. Anterior talocrural joint laxity: diagnostic accuracy of the anterior drawer test of the ankle. J Orthop Sports Phys Ther. 2013;43(12):911-9. PubMed PMID: rayyan-152988524.

109. de Vries JS, Kerkhoffs GM, Blankevoort L, van Dijk CN. Clinical evaluation of a dynamic test for lateral ankle ligament laxity. Knee Surg Sports Traumatol Arthrosc. 2010;18(5):628-33. PubMed PMID: rayyan-152988540.

110. Docherty CL, Gansneder BM, Arnold BL, Hurwitz SR. Development and Reliability of the Ankle Instability Instrument. Journal of Athletic Training (National Athletic Trainers' Association). 2006;41(2):154-8. PubMed PMID: rayyan-152988587.

111. Eechaute C, Leemans L, De Mesmaeker M, De Ridder R, Beckwée D, Struyf F, et al. The predictive value of the multiple hop test for first-time noncontact lateral ankle sprains. J Sports Sci. 2020;38(1):86-93. PubMed PMID: rayyan-152988595.

112. Fusco A, Giancotti GF, Fuchs PX, Wagner H, Varalda C, Cortis C. Wobble board balance assessment in subjects with chronic ankle instability. Gait Posture. 2019;68:352-6. PubMed PMID: rayyan-152988564.

113. Gabriel EH, Powden CJ, Hoch MC. Comparison of the Y-Balance Test and Star Excursion Balance Test: Utilization of a Discrete Event Simulation. J Sport Rehabil. 2020;30(2):214-9. Epub 20200423. doi: 10.1123/jsr.2019-0425. PubMed PMID: 32325427.

114. García-Rubio J, Pino J, Olivares PR, Ibáñez SJ. Validity and Reliability of the WIMU(TM) Inertial Device for the Assessment of Joint Angulations. Int J Environ Res Public Health. 2019;17(1). PubMed PMID: rayyan-152988590.

115. Gatt A, Chockalingam N. Validity and reliability of a new ankle dorsiflexion measurement device. Prosthet Orthot Int. 2013;37(4):289-97. PubMed PMID: rayyan-152988536.

116. Geerinck A, Beaudart C, Salvan Q, Van Beveren J, D'Hooghe P, Bruyère O, et al. French translation and validation of the Cumberland Ankle Instability Tool, an instrument for measuring functional ankle instability. Foot Ankle Surg. 2020;26(4):391-7. PubMed PMID: rayyan-152988591.

117. Ghorbani Faal S, Shirzad E, Sharifnezhad A, Ashrostaghi M, Naemi R. A Novel Method for Field Measurement of Ankle Joint Stiffness in Hopping. Applied Sciences. 2021;11(24):12140. PubMed PMID: doi:10.3390/app112412140.

118. Gourlay J, Bullock G, Weaver A, Matsel K, Kiesel K, Plisky P. The Reliability and Criterion Validity of a Novel Dorsiflexion Range of Motion Screen. ATHLETIC TRAIN SPORTS HEALTH CARE. 2020;12(1):40-4. PubMed PMID: rayyan-152988638.

119. Greve F, Braun KF, Vitzthum V, Zyskowski M, Müller M, Kirchhoff C, et al. The Munich Ankle Questionnaire (MAQ): a self-assessment tool for a comprehensive evaluation of ankle disorders. Eur J Med Res. 2018;23(1):46. PubMed PMID: rayyan-152988594.

120. Heitman RJ, Kovaleski JE, Pugh SF. Application of generalizability theory in estimating the reliability of ankle-complex laxity measurement. J Athl Train. 2009;44(1):48-52. PubMed PMID: rayyan-152988621.

121. Hiller CE, Refshauge KM, Bundy AC, Herbert RD, Kilbreath SL. The Cumberland ankle instability tool: a report of validity and reliability testing. Arch Phys Med Rehabil. 2006;87(9):1235-41. PubMed PMID: rayyan-152988525.

122. Hung M, Baumhauer JF, Licari FW, Bounsanga J, Voss MW, Saltzman CL. Responsiveness of the PROMIS and FAAM Instruments in Foot and Ankle Orthopedic Population. Foot Ankle Int. 2019;40(1):56-64. PubMed PMID: rayyan-152988626.

123. Hung M, Baumhauer JF, Licari FW, Voss MW, Bounsanga J, Saltzman CL. PROMIS and FAAM Minimal Clinically Important Differences in Foot and Ankle Orthopedics. Foot Ankle Int. 2019;40(1):65-73. PubMed PMID: rayyan-152988592.

124. Ibrahim T, Beiri A, Azzabi M, Best AJ, Taylor GJ, Menon DK. Reliability and validity of the subjective component of the American Orthopaedic Foot and Ankle Society clinical rating scales. J Foot Ankle Surg. 2007;46(2):65-74. PubMed PMID: rayyan-152988550.

125. Johanson NA, Liang MH, Daltroy L, Rudicel S, Richmond J, Johanson NA, et al. American Academy of Orthopaedic Surgeons lower limb outcomes assessment instruments. Reliability, validity, and sensitivity to change. J BONE JOINT SURG (AM). 2004;86(5):902-9. PubMed PMID: rayyan-152988645.

126. Jones R, Carter J, Moore P, Wills A, Jones R, Carter J, et al. A study to determine the reliability of an ankle dorsiflexion weight-bearing device. Physiotherapy. 2005;91(4):242-9. PubMed PMID: rayyan-152988655.

127. Kerkhoffs GMMJ, Blankevoort L, Sierevelt IN, Corvelein R, Janssen GHW, van Dijk CN. Two ankle joint laxity testers: reliability and validity. Knee Surgery, Sports Traumatology, Arthroscopy. 2005;13(8):699-705. PubMed PMID: rayyan-152988663.

128. Kleeblad LJ, van Bemmel AF, Sierevelt IN, Zuiderbaan HA, Vergroesen DA. Validity and Reliability of the Achillometer(®): An Ankle Dorsiflexion Measurement Device. J Foot Ankle Surg. 2016;55(4):688-92. PubMed PMID: rayyan-152988561.

129. Ko J, Rosen AB, Brown CN. Functional performance tests identify lateral ankle sprain risk: A prospective pilot study in adolescent soccer players. Scand J Med Sci Sports. 2018;28(12):2611-6. PubMed PMID: rayyan-152988589.

130. Liu K, Glutting J, Wikstrom E, Gustavsen G, Royer T, Kaminski TW. Examining the diagnostic accuracy of dynamic postural stability measures in differentiating among ankle instability status. Clin Biomech (Bristol, Avon). 2013;28(2):211-7. PubMed PMID: rayyan-152988633.

131. Martin RL, Irrgang JJ, Burdett RG, Conti SF, Van Swearingen JM. Evidence of validity for the Foot and Ankle Ability Measure (FAAM). Foot Ankle Int. 2005;26(11):968-83. PubMed PMID: rayyan-152988625.

132. Matheny LM, Clanton TO. Rasch Analysis of Reliability and Validity of Scores From the Foot and Ankle Ability Measure (FAAM). Foot Ankle Int. 2 ed. United States2020. p. 229-36.

133. Maurus P, Asmussen MJ, Cigoja S, Nigg SR, Nigg BM. The Submaximal Lateral Shuffle Test: A reliability and sensitivity analysis. J Sports Sci. 2019;37(18):2066-74. PubMed PMID: rayyan-152988528.

134. McGirr KA, Kennedy T, MÃ¸lgaard CM, Rathleff MS. Intra-Tester Reliability of Hand-Held Dynamometry and Strap-Mounted Dynamometry for Assessment of Ankle Strength. INT J ATHLETIC THER TRAIN. 2014;19(2):14-9. PubMed PMID: rayyan-152988643.

135. Menadue C, Raymond J, Kilbreath SL, Refshauge KM, Adams R. Reliability of two goniometric methods of measuring active inversion and eversion range of motion at the ankle. BMC Musculoskelet Disord. 2006;7:60. PubMed PMID: rayyan-152988627.

136. Meyer DC, Werner CML, Wyss T, Vienne P. A Mechanical Equinometer to Measure the Range of Motion of the Ankle Joint: Interobserver and Intraobserver Reliability. Foot Ankle Int. 2006;27(3):202-5. PubMed PMID: rayyan-152988666.

137. Nigg BM, Nigg CR, Reinschmidt C. Reliability and validity of active, passive and dynamic range of motion tests. Sportverletz Sportschaden. 1995;9(2):51-7. PubMed PMID: rayyan-152988609.

138. Petersen EJ, Irish SM, Lyons CL, Miklaski SF, Bryan JM, Henderson NE, et al. Reliability of water volumetry and the figure of eight method on subjects with ankle joint swelling. J Orthop Sports Phys Ther. 1999;29(10):609-15. PubMed PMID: rayyan-152988629.

139. Plisky PJ, Gorman PP, Butler RJ, Kiesel KB, Underwood FB, Elkins B. The reliability of an instrumented device for measuring components of the star excursion balance test. N Am J Sports Phys Ther. 2009;4(2):92-9. PubMed PMID: rayyan-152988635.

140. Rodríguez-Fernández Á L, Rebollo-Roldán J, Jiménez-Rejano JJ, Güeita-Rodríguez J. Psychometric properties of the Spanish version of the Cumberland Ankle Instability Tool. Disabil Rehabil. 2015;37(20):1888-94. PubMed PMID: rayyan-152988598.

141. Smith DH, Hoch JM, Facchini SJ, Hoch MC. Intra-rater and Inter-rater Reliability and Responsiveness of the Posterior Talar Glide Test. ATHLETIC TRAIN SPORTS HEALTH CARE. 2015;7(1):23-8. PubMed PMID: rayyan-152988640.

142. Smith MD, Lee D, Russell T, Matthews M, MacDonald D, Vicenzino B. How Much Does the Talocrural Joint Contribute to Ankle Dorsiflexion Range of Motion During the Weight-Bearing Lunge Test? A Cross-sectional Radiographic Validity Study. J Orthop Sports Phys Ther. 2019;49(12):934-41. PubMed PMID: rayyan-152988618.

143. Vela LI, Denegar CR, Vela LI, Denegar CR. The Disablement in the Physically Active Scale, Part II: The Psychometric Properties of an Outcomes Scale for Musculoskeletal Injuries. J Athl Train. 2010;45(6):630-41. PubMed PMID: rayyan-152988580.

144. Vohralik SL, Bowen AR, Burns J, Hiller CE, Nightingale EJ. Reliability and validity of a smartphone app to measure joint range. Am J Phys Med Rehabil. 2015;94(4):325-30. PubMed PMID: rayyan-152988534.

145. Vuurberg G, Kluit L, van Dijk CN. The Cumberland Ankle Instability Tool (CAIT) in the Dutch population with and without complaints of ankle instability. Knee Surg Sports Traumatol Arthrosc. 2018;26(3):882-91. PubMed PMID: rayyan-152988608.

146. Wilken J, Rao S, Estin M, Saltzman CL, Yack HJ. A new device for assessing ankle dorsiflexion motion: reliability and validity. J Orthop Sports Phys Ther. 2011;41(4):274-80. PubMed PMID: rayyan-152988545.

147. Youdas JW, Bogard CL, Suman VJ. Reliability of goniometric measurements and visual estimates of ankle joint active range of motion obtained in a clinical setting. Arch Phys Med Rehabil. 1993;74(10):1113-8. PubMed PMID: rayyan-152988624.

148. Angthong C, Chernchujit B, Suntharapa T, Harnroongroj T. Visual analogue scale foot and ankle: validity and reliability of Thai version of the new outcome score in subjective form. J Med Assoc Thai. 2011;94(8):952-7. PubMed PMID: rayyan-152988614.

149. Arunakul M, Arunakul P, Suesiritumrong C, Angthong C, Chernchujit B. Validity and Reliability of Thai Version of the Foot and Ankle Ability Measure (FAAM) Subjective Form. J Med Assoc Thai. 2015;98(6):561-7. PubMed PMID: rayyan-152988619.

150. Borloz S, Crevoisier X, Deriaz O, Ballabeni P, Martin RL, Luthi F. Evidence for validity and reliability of a French version of the FAAM. BMC Musculoskelet Disord. 2011;12(100968565):40. PubMed PMID: rayyan-152988651.

151. Boszczyk A, Błoński M, Pomianowski S. Translation, Cultural Adaptation and Validation of Polish Version of Foot and Ankle Outcomes Questionnaire. Ortop Traumatol Rehabil. 2015;17(2):175-87. PubMed PMID: rayyan-152988605.

152. Çelik D, Malkoç M, Martin R. Evidence for reliability, validity and responsiveness of Turkish Foot and Ankle Ability Measure (FAAM). Rheumatol Int. 2016;36(10):1469-76. PubMed PMID: rayyan-152988604.

153. Haji-Maghsoudi M, Naseri N, Nouri-Zadeh S, Jalayi S, Haji-Maghsoudi M, Naseri N, et al. Evidence of Reliability for Persian Version of the "Cumberland Ankle Instability Tool (CAIT)" in Iranian Athletes with lateral Ankle Sprain. Arch Rehabil. 2016;16(4):304-10. PubMed PMID: rayyan-152988652.

154. Imoto AM, Peccin MS, Rodrigues R, Mizusaki JM, Imoto AM, Peccin MS, et al. TRANSLATION, CULTURAL ADAPTATION AND VALIDATION OF FOOT AND ANKLE OUTCOME SCORE (FAOS) QUESTIONNAIRE INTO PORTUGUESE. Acta Ortop Bras. 2009;17(4):232-5. PubMed PMID: rayyan-152988654.

155. Karatepe AG, Günaydin R, Kaya T, Karlibaş U, Özbek G. Validation of the Turkish version of the foot and ankle outcome score. Rheumatol Int. 2009;30(2):169-73. PubMed PMID: rayyan-152988602.

156. Kim JB, Kim JK, Seo SG, Lee DY. Validity, reliability, and responsiveness of the Korean version of American Academy of Orthopedic Surgeons Foot and Ankle questionnaire. J Foot Ankle Surg. 2015;54(1):46-50. PubMed PMID: rayyan-152988610.

157. Lee KM, Chung CY, Kwon SS, Sung KH, Lee SY, Won SH, et al. Transcultural adaptation and testing psychometric properties of the Korean version of the Foot and Ankle Outcome Score (FAOS). Clin Rheumatol. 2013;32(10):1443-50. PubMed PMID: rayyan-152988613.

158. Ling SKK, Chan V, Ho K, Ling F, Lui TH. Reliability and validity analysis of the open-source Chinese Foot and Ankle Outcome Score (FAOS). Foot (Edinb). 2018;35:48-51. PubMed PMID: rayyan-152988620.

159. Mazaheri M, Salavati M, Negahban H, Sohani SM, Taghizadeh F, Feizi A, et al. Reliability and validity of the Persian version of Foot and Ankle Ability Measure (FAAM) to measure functional limitations in patients with foot and ankle disorders. Osteoarthritis Cartilage. 2010;18(6):755-9. PubMed PMID: rayyan-152988615.

160. Mineta S, Inami T, Fukano M, Hoshiba T, Masuda Y, Yoshimura A, et al. The reliability, and discriminative ability of the identification of functional ankle instability questionnaire, Japanese version. Phys Ther Sport. 2019;35:1-6. PubMed PMID: rayyan-152988593.

161. Mousavian A, Ebrahimzadeh MH, Birjandinejad A, Omidi-Kashani F, Kachooei AR. Translation and cultural adaptation of the Manchester-Oxford Foot Questionnaire (MOXFQ) into Persian language. FOOT. 2015;25(4):224-7. PubMed PMID: rayyan-152988639.

162. Negahban H, Mazaheri M, Salavati M, Sohani SM, Askari M, Fanian H, et al. Reliability and validity of the foot and ankle outcome score: a validation study from Iran. Clin Rheumatol. 2010;29(5):479-86. PubMed PMID: rayyan-152988607.

163. Sierevelt IN, Beimers L, van Bergen CJA, Haverkamp D, Terwee CB, Kerkhoffs G. Validation of the Dutch language version of the Foot and Ankle Outcome Score. Knee Surg Sports Traumatol Arthrosc. 2015;23(8):2413-9. PubMed PMID: rayyan-152988600.

164. Uematsu D, Suzuki H, Sasaki S, Nagano Y, Shinozuka N, Sunagawa N, et al. Evidence of Validity for the Japanese Version of the Foot and Ankle Ability Measure. Journal of Athletic Training (Allen Press). 2015;50(1):65-70. PubMed PMID: rayyan-152988664.

165. van Bergen CJ, Sierevelt IN, Hoogervorst P, Waizy H, van Dijk CN, Becher C. Translation and validation of the German version of the foot and ankle outcome score. Arch Orthop Trauma Surg. 2014;134(7):897-901. PubMed PMID: rayyan-152988601.

166. Venditto T, Tognolo L, Rizzo RS, Iannuccelli C, Di Sante L, Trevisan M, et al. 17-Italian Foot Function Index with numerical rating scale: development, reliability, and validity of a modified version of the original Foot Function Index. Foot (Edinb). 2015;25(1):12-8. PubMed PMID: rayyan-152988612.

167. Weel H, Zwiers R, Azim D, Sierevelt IN, Haverkamp D, van Dijk CN, et al. Validity and reliability of a Dutch version of the Foot and Ankle Ability Measure. Knee Surg Sports Traumatol Arthrosc. 2016;24(4):1348-54. PubMed PMID: rayyan-152988599.

168. Yazıcı G, Yazıcı MV, Bayraktar D, Varol F, Güçlü Gündüz A, Bek N. Validity and reliability of the Turkish version of the Self-reported Foot and Ankle Score in patients with foot or ankle pain. Acta Orthop Traumatol Turc. 2020;54(4):408-13. PubMed PMID: rayyan-152988628.

169. Doherty C, Bleakley C, Hertel J, Caulfield B, Ryan J, Delahunt E. Clinical Tests Have Limited Predictive Value for Chronic Ankle Instability When Conducted in the Acute Phase of a First-Time Lateral Ankle Sprain Injury. Arch Phys Med Rehabil. 2018;99(4):720-5.e1. PubMed PMID: rayyan-666947566.

170. Eechaute C, Vaes P, Duquet W, Van Gheluwe B. Reliability and discriminative validity of sudden ankle inversion measurements in patients with chronic ankle instability. England2009 2009-7. 82-6 p.

171. Hartley E, Hoch M, McKeon P. Reliability and responsiveness of gait initiation profiles in those with chronic ankle instability. Gait Posture. 2016;49:86-9. PubMed PMID: rayyan-666947564.

172. Hubbard TJ, Kramer LC, Denegar CR, Hertel J. Correlations among multiple measures of functional and mechanical instability in subjects with chronic ankle instability. Journal of Athletic Training (National Athletic Trainers' Association). 2007;42(3):361-6. PubMed PMID: rayyan-666947582.

173. Johnson MR, Stoneman PD. Comparison of a lateral hop test versus a forward hop test for functional evaluation of lateral ankle sprains. J Foot Ankle Surg. 3 ed. United States2007. p. 162-74.

174. Kobayashi T, Mizota T, Kon K, Kasaya M, Miyabe S, Shindome T, et al. The Reliability and Validity of a Novel Ankle Isometric Plantar Flexion Strength Test. J Sport Rehabil. 2022:1-7. Epub 20220208. doi: 10.1123/jsr.2021-0192. PubMed PMID: 35135897.

175. Lindstrand A, Mortensson W, Norman O. Talofibular compartment of the ankle joint after recent ankle sprain. Acta Radiol Diagn (Stockh). 1978;19(5):847-52. Epub 1978/01/01. doi: 10.1177/028418517801900514. PubMed PMID: 717035.

176. Lohrer H, Nauck T, Gehring D, Gollhofer A. [Ankle arthrometry for evaluation of the mechanical component in chronic ankle instability]. Sportverletz Sportschaden. 2013;27(2):85-90. PubMed PMID: rayyan-666947569.

177. Park YH, Park SH, Kim SH, Choi GW, Kim HJ, Park YH, et al. Relationship Between Isokinetic Muscle Strength and Functional Tests in Chronic Ankle Instability. J Foot Ankle Surg. 2019;58(6):1187-91. PubMed PMID: rayyan-666947560.

178. Rosen A, Ko J, Brown C. A Multivariate Assessment of Clinical Contributions to the Severity of Perceived Dysfunction Measured by the Cumberland Ankle Instability Tool. Int J Sports Med. 2016;37(14):1154-8. PubMed PMID: rayyan-666947559.

179. Terrier R, Degache F, Fourchet F, Gojanovic B, Forestier N, Terrier R, et al. Assessment of evertor weakness in patients with chronic ankle instability: Functional versus isokinetic testing. Clin Biomech. 2017;41:54-9. PubMed PMID: rayyan-666947588.

180. Theurillat C, Punt I, Armand S, Bonnefoy-Mazure A, Allet L. Active Ankle Circumduction to Identify Mobility Deficits in Subacute Ankle Sprain Patients. Journal of applied biomechanics. 2018;34(1):1-6. PubMed PMID: rayyan-152988535.

181. Udompanich N, Thanasootr KO, Chanavirut R, Chatchawan U, Hunsawong T. The Cut-Off Score of Four Clinical Tests to Quantify Balance Impairment in Individuals with Chronic Ankle Instability. Malays J Med Sci. 2021;28(4):87-96. Epub 20210826. doi: 10.21315/mjms2021.28.4.9. PubMed PMID: 34512133; PubMed Central PMCID: PMCPMC8407791.

182. Chiu YL, Tsai YJ, Lin CH, Hou YR, Sung WH, Chiu Y-L, et al. Evaluation of a smartphone-based assessment system in subjects with chronic ankle instability. Comput Meth Programs Biomed. 2017;139:191-5. PubMed PMID: rayyan-666947587.

183. Dickson D, Hollman K, Bronner S, Ojofeitimi S. Reliability, validity, and interpretation of a new way to measure ankle dorsiflexion...2008 Combined Sections Meeting...Nashville, Tennessee, February 6-9, 2008. Journal of Orthopaedic & Sports Physical Therapy. 2008;38(1):A20-A. PubMed PMID: rayyan-152988650.

184. Lavery LA, Armstrong DG. ACFAS scoring scale: ready, fire, aim? J Foot Ankle Surg. 2006;45(4):284-5; author reply 5-6. PubMed PMID: rayyan-152988622.

185. Linens SW. Determining sensitive and accurate measures for detecting balance deficits associated with functional ankle instability: Virginia Commonwealth University; 2009.

186. Alcock GK, Stratford PW. Validation of the Lower Extremity Functional Scale on athletic subjects with ankle sprains. PHYSIOTHER CAN. 2002;54(4):233-40. PubMed PMID: rayyan-152988572.

187. Nistor L, Markhede G, Grimby G. A technique for measurements of plantar flexion torque with the Cybex II dynamometer. Scand J Rehabil Med. 1982;14(4):163-6. PubMed PMID: rayyan-152988634.

188. De Noronha M, Hiller CE, Nightingale EJ, Refshauge KM. Validity and reliability of a single-limb balance test. Journal of Orthopaedic & Sports Physical Therapy. 2009;39(10):A21-A. PubMed PMID: rayyan-152988644.

189. Hiller CE, Bundy A, Killbreath SL, Refshauge KM. Generalizability of the Cumberland Ankle Instability Tool (CAIT). Journal of Orthopaedic & Sports Physical Therapy. 2009;39(10):A21-A. PubMed PMID: rayyan-152988648.
