# Supplementary material for: Impairment-based assessments for patients with lateral ankle sprain: A systematic review of measurement properties
Source: PLoS One. 2023 Feb 22;18(2):e0280388. doi: 10.1371/journal.pone.0280388 (PMC9946235; doi:10.1371/journal.pone.0280388)
Supplement: Attachment — Submitted filename: Response to Reviewers_PlosOne.pdf [file pone.0280388.s004.pdf]

Response to the reviewers for publication in PlosOne

Adaptions made for the journal requirements

a) **Adapted the financial disclosure section**

The sentence for the financial disclosure was adapted to the requirements of PlosOne for a study without financial support (p.3). This section was also added in the Cover Letter.

b) **Competing interests' section**

This was also adapted to the requirements of PlosOne in both the manuscript (p.3) and the cover letter.

Adaptions made based on first reviewer`s feedback

Dear Mr Craig Denegar,
Thank you for your feedback on our systematic review. We have adapted the manuscript with your feedback. A detailed description is added below:

a) **Line 104**

The aspect "data were" was adapted.

b) **Line 202, respectively 200-203**

Adapted the section accordingly (line 200 to 203). As most of the population (n=9 of 15) were assessed within four weeks since injury, we allocated the Mawdsley study to the acute population. Therefore, the following sections were adapted:

- Abstract, Result section: now 10 studies in acute setting, 39 in history of lateral ankle sprain (LAS) population
- Line 174: now 10 studies in acute setting
- Line 175: now 39 studies in history of LAS population
- Line 209, Table 4: adapted the heading of the swelling study to ≤4 weeks prior
- Line 218, Table 6: adapted the heading of the swelling study to ≤4 weeks prior

c) **Line 251**

The stated percentage was adapted to 5 to 93%.

d) **Line 398-400**

The sentences in line 398-400 regarding ultrasonography and its recommendation for the identification of LAS were adapted and clarified.

e) **Line 459**

The sentence on the inflammatory response was adapted as recommended.

Adaptions made based on the second reviewer`s feedback

Dear second reviewer,
Thank you for your detailed feedback regarding our systematic review. The manuscript is now adapted according to your feedback. Please find attached a description of our adaptions.

a) **Check editorial rules and other editorial aspects**

The manuscript was adapted based on the PlosOne guidelines. This included for example the adaption of the references to the Vancouver style (p.53-60), of the Abstract (p.2), headings and figure and table referencing (line 56, 168, 169, 184, 190, 202, 239, 246, 281, 302, 315, 337).

b) **Manuscript correction by a person of English mother tongue**

The manuscript was checked by a person of English mother tongue.

c) **Abstract**

Type of the study, research timing and study settings was added to the methods section in the abstract (p.2.). The structure of the abstract was adapted to the guidelines of PlosOne.

d) **Key words**

The key words were provided in alphabetic order and new key words were added (p.2).

e) Background

The hypothesis of this study is described in more detail (line 24 to 28).
It was mentioned that the reference for the anatomical details of the ankle joint should be added to the manuscript. Moreover, the second reviewer wished to include more information about the medial and lateral compartment of the ankle joint. Since lateral ankle sprains primarily injure the lateral ligamentous apparatus, we would refrain from providing information on the medial ligamentous apparatus and specifically the deltoid ligament. In addition, in the context of the systematic review, the impairments due to the lateral ankle sprain are in the focus, and for us this should be primarily emphasised in the introduction. Therefore, we have not yet referenced the study and have not included a section describing the ligament structures in more detail. However, we are open to feedback or information on why these aspects and specifically this study should be included.

f) Methods

The exact period of study selection was added: line 66, 71, 72
Thank you for your feedback regarding the risk of bias. This is a highly relevant aspect. When reading our systematic review again, we have mentioned the different risk of bias tools that we have used and dimensions that were assessed (line 95 to 119). Additionally, we have added aspects on what we – as authors – have done to reduce risk of bias such as reporting or selection bias (line 75-76, line 79, line 96-97, line 158-159). We have added a sentence in line 131 to 132 regarding assessment of the measurement properties. If this is still not sufficient, please indicate which type of study bias assessment needs to be described in more detail.

g) Statistical analysis

The statistical analysis method was specified in lines 160 to 161.

h) Results

The results section was now adapted to a more readable fashion. Therefore, page 11 is now filled with all written aspects. In the following, all tables (Tables 2-6) referenced there come one after the other in the following pages (page 12-35). When proofreading the final document produced by the journal, we check the manuscript again for readability.

i) Discussion

First, we acknowledge your feedback regarding the inclusion the systematic review and meta-analysis on kinesio taping and the effect on sport performance and ankle function in athletes with chronic ankle instability. The main statement of this systematic review is how kinesio taping could influence gait, range of motion, muscle activation and postural sway. Our research group has primarily focussed on the assessments of impairments and less on interventions. We would therefore rather refrain from including the study. However, you are welcome to tell us why including this specific study would be important for our study manuscript and its story telling.

The aspects regarding bias and weakness of the study were specified (lines 499-501, line 505-506).

j) References

The references were adapted using the Vancouver style. Studies that were not relevant and were published before 2010 were replaced. This applies especially to the Verhagen study on the cost of illness of a lateral ankle sprain. It was replaced by the Bielska study. Other studies could not be replaced as they were part of the studies included in the systematic review.

Thank you for the input on the Systematic review on impairment-based testing in lateral ankle sprain patients.
We hope that the changes are as expected.
